# Wearable Sensors in Gait Assessment for Parkinson Disease and Stroke in Real-World Environments: Systematic Review and Meta-Analysis

**DOI:** 10.2196/93413

**Published:** 2026-09-25

**Authors:** Saskia Neumann, Jose Albites-Sanabria, Aileen C Naef, Andreas R Luft, Lorenzo Chiari, Chris Easthope Awai

**Affiliations:** 1 Data Analytics & Rehabilitation Technology (DART) at Lake Lucerne Institute, Rubistrasse 9, Vitznau, 6354, Switzerland, 41 77 466 8135; 2 Faculty of Medicine, University of Zurich, Zurich, Switzerland; 3 Department of Electrical, Electronic and Information Engineering "Guglielmo Marconi", University of Bologna, Bologna, Italy; 4 Rehabilitation Engineering Laboratory, ETH Zurich, Zurich, Switzerland; 5 Department of Neuology, University of Zurich and University Hospital Zurich, Zurich, Switzerland; 6 cereneo Center for Neurology and Rehabilitation, Vitznau, Switzerland; 7 Lake Lucerne Institute, Vitznau, Switzerland; 8 Health Sciences and Technologies, Interdepartmental Center for Industrial Research (CIRI-SDV), University of Bologna, Bologna, Italy

**Keywords:** accelerometer, gait assessment, Parkinson disease, real-world, remote monitoring, stroke, wearable sensors

## Abstract

**Background:**

Real-world gait assessment has gained momentum in populations with walking impairments, offering insights beyond standardized tests and supporting the integration of remote monitoring into clinical care. However, its potential remains limited by the lack of validated population- and context-specific digital biomarkers.

**Objective:**

The primary objective was to summarize and critically evaluate the current state of real-world gait assessment using wearable sensors in Parkinson disease (PD) and stroke, addressing methodological approaches, sensor configurations, validation strategies, and outcome reporting. The secondary objective was to quantify methodological heterogeneity by reporting pooled means with 95% confidence intervals (CIs) and prediction intervals (PIs) of commonly reported gait parameters.

**Methods:**

PubMed, Scopus, Web of Science, and Embase were searched for English-language studies published up to April 20, 2026. Eligible studies used wearable sensors to assess gait quality parameters in real-world settings in at least 5 individuals with PD or poststroke. Laboratory- or rehabilitation-only studies, non–peer-reviewed articles, abstracts, and studies before 2014 were excluded. Methodological quality was assessed with a checklist adapted from Hubble et al. Gait features available in at least 5 reports were included in a meta-analysis using a random-effects model with Hartung-Knapp-Sidik-Jonkman adjustment, deriving 95% CIs and PIs to characterize the pooled estimate and its dispersion.

**Results:**

Of 2489 records, 43 reports were included: 37 on PD (2774 participants; mean age 67.89, SD 8.68 years) and 6 on stroke (217 participants; mean age 63.83, SD 11.84 years). Methodological quality was high, but external validity was consistently the weakest domain in both populations. Sensor configurations most commonly consisted of a lower back accelerometer. In total, 13 distinct walking bout definitions were identified, whereas 17 reports provided none, indicating substantial terminological heterogeneity. In PD, 4 gait parameters met the meta-analysis threshold, each showing a PI far wider than its CI, precluding generalization to future settings. For example, gait speed pooled at 0.84 m/s (95% CI 0.77‐0.91; 95% PI 0.56‐1.12), and number of steps per day at 7202 (95% CI 3975‐10,429), the latter with a PI extending below zero, a mathematically impossible range reflecting extreme dispersion. No stroke parameter was reported consistently enough to permit meta-analysis, underscoring a critically underdeveloped evidence base.

**Conclusions:**

Unlike previous reviews focusing on single diseases or specific hardware, this review contrasts PD and stroke to highlight shared methodological challenges. Real-world gait assessment in PD is advancing but fragmented, whereas the stroke evidence base remains sparse. The observed variance is compounded by procedural and algorithmic inconsistencies that cannot currently be separated from genuine clinical differences. The methodological groundwork established in PD offers a foundation for early adoption of standardized terminology, validation procedures, and core outcomes, essential for transitioning wearable sensors from exploratory tools to reliable clinical and remote-monitoring instruments.

## Introduction

Neurological disorders are the leading cause of disability-adjusted life-years and a major contributor to global disability, requiring long-term access to care and treatment, monitoring, and rehabilitation to improve patient outcomes and quality of life [1,2]. Among these conditions, Parkinson disease (PD) is the fastest-growing neurodegenerative disorder, while stroke remains the leading cause of long-term disability worldwide [3]. Despite differing pathophysiology, both conditions share a clinically important consequence, namely, characteristic gait disturbances that substantially impair mobility in daily life and rank among the most prevalent contributors to reduced quality of life and functional independence [4-6]. In PD, gait is typically marked by freezing episodes, reduced gait speed, shorter step length, prolonged double-support time, and increased stride-to-stride variability [7,8]. Poststroke gait, in contrast, is characterized by persistent asymmetry, reduced gait speed, and altered step patterns that may limit recovery and community reintegration [9]. Beyond these phenotypic differences, both populations share a fundamental clinical challenge: the reliable and meaningful assessment of gait in everyday life. In PD and stroke, gait impairments are chronic, multifactorial, and subject to temporal fluctuations, shaped by a complex interplay of individual, task-related, and environmental factors that isolated point-in-time clinical assessments cannot fully capture [10-13].

This challenge is formally recognized in the World Health Organization’s International Classification of Functioning, Disability and Health, which differentiates between 2 constructs central to understanding functional ability [14]. Capacity refers to an individual’s maximal functioning in a standardized or controlled setting, whereas performance reflects how that individual actually moves within their natural, everyday environment [15]. Assessing both constructs provides valuable and complementary clinical insight; yet, current practice focuses predominantly on capacity, leaving performance systematically underassessed [10]. Wearable sensor technologies address this gap directly by enabling continuous, unobtrusive monitoring under real-world conditions, shifting the focus from what patients can do in the clinic to what they actually do in daily life, thereby enhancing the ecological validity of gait assessment and supporting clinical decision-making [16-18].

Early wearable-based approaches relied primarily on pedometers and activity trackers, capturing step counts, walking distance, and activity bouts [19]. While informative about overall physical activity levels, these measures cannot characterize gait quality. Features such as spatiotemporal variability, asymmetry, coordination, and disease-specific motor symptoms may be more sensitive to motor dysfunction than traditional clinical rating scales; yet, they remain beyond the reach of simple activity monitors [20,21]. This is reflected in a systematic review of 30 studies on walking performance after stroke, in which the most commonly reported outcomes were step counts, step timing, walking distance, and walking bouts [22]. Inertial measurement units (IMUs) represent a meaningful advance in this regard, enabling the extraction of richer, more granular gait outcomes under real-world conditions. Studies have shown that IMU-derived digital mobility outcomes can distinguish people with PD from healthy controls and differentiate real-world from supervised walking, underscoring their clinical potential [23]. Recent advances include algorithms tailored to patient-specific characteristics and validation approaches that extend beyond simple laboratory tasks [24,25]. However, important challenges remain. Accurately assessing gait in severely impaired individuals is particularly difficult in the presence of slow gait speed, assistive device use, frequent breaks, and short walking bouts [24,26-28]. Broader methodological barriers, including inconsistent data collection protocols, lack of standardization, limited algorithm validation, and unclear clinical relevance of many sensor-derived outcomes, further limit progress in the field.

Existing reviews have only partially addressed these challenges. In PD, reviews have largely focused on wearable technologies for motor symptom detection and management, the identification of digital biomarkers for remote monitoring, or specific sensor configurations and data-processing approaches [23,29-33]. In stroke, recent reviews have centered predominantly on physical activity monitoring or rehabilitation-oriented assessment frameworks [34-37]. To our knowledge, no review has yet systematically examined wearable-based real-world gait assessment across both populations, despite their shared assessment challenges and the growing clinical need for validated, ecologically meaningful outcomes. As the field develops rapidly, a timely synthesis is needed to clarify the current state of the evidence and highlight where methodological and terminological heterogeneity remain pronounced. This systematic review and meta-analysis therefore evaluates the current state of wearable sensor-based real-world gait assessment in PD and stroke, examining sensor setups, IMU-derived outcomes, validation strategies, and methodological approaches, while identifying the key barriers and areas where greater consensus will be essential for future progress. Where gait features were available in at least 5 reports, a meta-analysis was conducted to quantitatively synthesize the available evidence and characterize the range of values reported across the literature.

## Methods

### Protocol and Registration

This systematic review and meta-analysis was registered with PROSPERO (International Prospective Register of Systematic Reviews: CRD42024531665). It was conducted in accordance with the PRISMA (Preferred Reporting Items for Systematic Reviews and Meta-Analyses) 2020 guidelines, including the PRISMA 2020 for Abstracts checklist [38], the PRISMA-S (Preferred Reporting Items for Systematic Reviews and Meta-Analyses Literature Search Extension) 2021 guidelines [39], and the framework outlined by Khan et al [40]. All checklists are provided in Checklists 1-3.

### Search Strategy and Selection Criteria

A systematic search in PubMed, Scopus, Web of Science, and Embase was performed for English-language studies published up to April 20, 2026. The reference lists of included studies and previous systematic reviews were screened for additional potentially eligible studies. No supplementary search methods, such as gray literature sources or expert contacts, were used. The following terms were searched as keywords: “Parkinson’s disease,” “stroke,” “gait,” “accelerometry,” “wearable sensor,” and “real-world.” The comprehensive search string for each database is provided in Multimedia Appendix 1. Two independent reviewers (SN and JA-S) screened all records at each stage of the selection process, including title, abstract, and full-text assessment. Disagreements were resolved by consensus or, when needed, by a third reviewer (ACN). Authors were contacted for clarification of study details when necessary.

Eligibility criteria were based on the population, intervention, comparison, outcomes, and study design framework [41]. Eligible studies enrolled at least 5 individuals with PD or stroke and used body-worn sensors to assess and report gait quality parameters in real-world settings. Studies were excluded if gait assessments were conducted solely in laboratory or rehabilitation settings, or if they were systematic reviews, non–peer-reviewed articles, or abstracts. The search was restricted to studies published from 2014 onward, as advances in sensor miniaturization, battery life, and data transmission around this period substantially increased the feasibility of prolonged gait monitoring in daily-life settings. Articles were also considered ineligible if they did not report extractable real-world gait outcomes of interest. Studies presenting only classification results, prediction scores, or model performance metrics without corresponding gait parameters were therefore excluded.

### Data Extraction and Analysis

Screening and data extraction were performed using Covidence (Veritas Health Innovation) templates to ensure consistency. One reviewer (SN) extracted the following information: publication details (title, year, first author, and country), study characteristics (sample size and clinical concept of interest), participant characteristics (age, sex, and diagnosis), data and algorithm origin and accessibility (study location and availability of data and algorithms), study setting (real-world or combined laboratory and real-world), methods (monitoring duration, sensor type and placement, and algorithm validation), gait-related outcome parameters (macro and micro), and methodological details (study aim and walking bout definition). All extracted data were checked by a second reviewer (JA-S), and discrepancies were resolved by consensus or, when necessary, by a third reviewer (ACN). Missing or unclear information was marked as “not available” if it was not directly stated in the report or could not be clearly traced or verified from the cited references.

Gait parameters were categorized into macro and micro characteristics [42]. Macro gait characteristics describe higher-level daily ambulatory patterns, including volume and variability measures such as total walking time per day, number and duration of walking bouts, and total step count, as well as advanced nonlinear metrics such as the power-law distribution (α) and within-bout variability (S^2^) [25,43,44]. Micro gait characteristics focus on spatiotemporal parameters, including their variability and asymmetry, within individual walking bouts. Studies meeting inclusion criteria were synthesized narratively, and a meta-analysis of data collected in real-world settings was performed when at least 5 eligible reports provided comparable gait parameters from distinct populations to quantitatively characterize the range of values reported across the literature.

### Methodological Quality

Methodological quality was assessed independently by 2 reviewers (SN and JA-S) using a checklist adapted from Hubble et al [45], which evaluates studies across 4 domains: reporting, external validity, internal validity, and confounding and selection bias. As the original checklist was designed for interventional and cohort study designs, it was adapted to fit the observational nature of the reports included in this review. This resulted in a modified 18-item version in which 8 items were reworded or added to better align the tool with this review’s focus on real-world monitoring and algorithm reporting (Multimedia Appendix 2). Specifically, in the reporting domain, 5 of the 10 original items were retained, and 5 were reworded. In the external validity domain, 2 of the 3 original items were retained, and 2 were added. In the internal validity domain, 3 of the 7 original items were retained, and 1 was added. The original 6-item confounding and selection bias domain was excluded entirely, as its content was specific to cohort study methodology and was not relevant to the included reports. The adapted checklist was piloted prior to full application. Disagreements in quality scoring were resolved by consensus or, when necessary, by a third reviewer (ACN). Quality scores were expressed as percentages and used to descriptively characterize the methodological quality of the included reports. Given the heterogeneity of study designs and the exploratory nature of the review, quality scores were not used to exclude reports or to weight the meta-analysis.

### Meta-Analysis Methods

When reports provided data from both laboratory and real-world settings, only real-world data were extracted and included in the quantitative synthesis. To avoid double counting in the meta-analysis, each dataset was included only once per outcome. When multiple reports were based on the same dataset, the report with the largest number of participants was selected. When outcomes were reported separately for distinct subgroups, such as fallers and nonfallers, subgroup data were first combined and treated as a single study sample. When outcomes were reported at multiple time points, the time point with the largest sample size was used. Prior to quantitative pooling, strict data extraction rules were applied to address clinical and methodological heterogeneity to prevent statistical distortion.

Reports were excluded from the meta-analysis and reserved for qualitative synthesis only if they met either of the following criteria: (1) fragmented bout definitions, whereby parameters were presented only in disjointed subcategories without providing an overall aggregate mean for steady-state walking (eg, isolating 10‐ to 60-second bouts from >60-second bouts); and (2) incompatible temporal units, whereby macro gait parameters were reported in scaled time frames such as steps per hour, as extrapolating these to daily totals introduces artificial variance, given the nonuniform nature of daily ambulation. Prior to pooling, all extracted parameters were harmonized to standard units (eg, meters per second for gait speed, steps per minute for cadence, meters for spatial parameters, and seconds for temporal parameters). For each included report, the mean, SD, and variance of gait parameters were extracted or computed. When reports provided medians and IQRs, the SD was estimated as SD=(p75–p25)/1.349. This conversion was required in 3 reports for gait speed and 2 reports for cadence. While this formula assumes approximate normality, it allowed these datasets to be included in the quantitative synthesis. A random-effects model was selected a priori, as the true effect size was expected to vary across studies due to differences in sensor configurations, gait detection algorithms, and population characteristics. To provide a more conservative estimation of the uncertainty around the pooled averages, the Hartung-Knapp-Sidik-Jonkman adjustment was applied. Between-study variance (τ²) was estimated using the restricted maximum likelihood method. In addition to 95% confidence intervals (CIs), 95% prediction intervals (PIs) were calculated to quantify the expected range of true effects in future real-world assessments. Formal assessments of small-study effects (eg, funnel plot asymmetry) were not performed because of the small number of reports available per parameter. In addition, according to Cochrane guidelines, such tests are not recommended in the presence of extreme between-study heterogeneity, reflected here in PIs far wider than the corresponding CIs, as any observed asymmetry likely reflects clinical and methodological variation rather than true publication bias [46]. All analyses were conducted in R (version 4.5; R Foundation for Statistical Computing) using RStudio and the *meta* package (version 8.3).

### Certainty of Evidence

The certainty of evidence was assessed for each meta-analyzed outcome using the GRADE (Grading of Recommendations Assessment, Development, and Evaluation) approach. As all evidence was observational, certainty started at low and was evaluated across 5 domains. Risk of bias was judged using a modified quality score (mean of the reporting and internal-validity domain scores from Multimedia Appendix 2); external validity was excluded to avoid double-counting with the indirectness domain. Reports scoring below 75% on this modified score were classified as having relevant concerns, and risk of bias was rated serious if such reports contributed at least half of the pooled weight for an outcome. Inconsistency was based on PI width relative to the CI [47]. Indirectness was based on participant representativeness and algorithm validation setting. Imprecision was based on the relative width of the 95% CI around the pooled mean, with a threshold of 30% for a serious rating. Funnel-plot asymmetry testing was not performed, as k<10 for 2 outcomes, and very wide PIs render asymmetry uninterpretable for the remaining 2. Assessments were performed independently by 2 reviewers (SN and JA-S), with disagreements resolved by consensus.

## Results

### Study Selection and Characteristics

**Figure 1.**
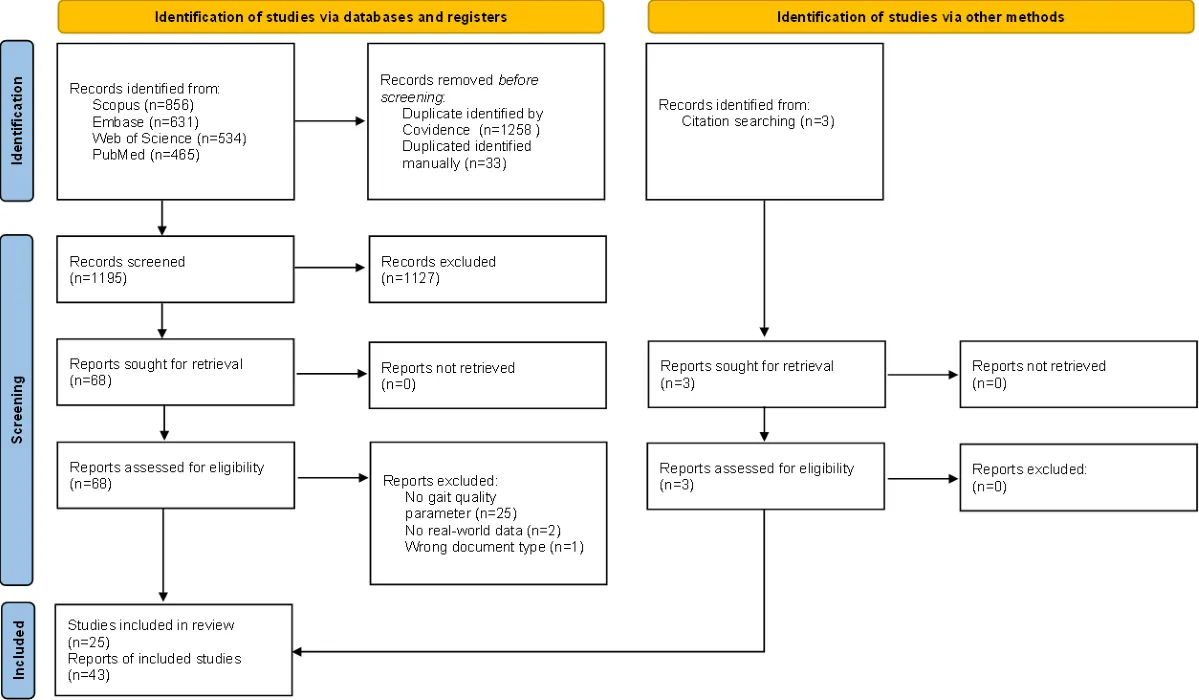
The PRISMA (Preferred Reporting Items for Systematic Reviews and Meta-Analyses) flow diagram of the study selection process.

The search identified 2486 records, with 3 additional records identified through manual reference checks. After duplicate removal and title and abstract screening, 71 reports were selected for full-text review. Of these, 43 reports met the inclusion criteria, comprising 37 reports on PD (2774 cumulative participants; mean age 67.89, SD 8.68 years) [24,26-28,43,44,48-78] and 6 reports on stroke (217 cumulative participants; mean age 63.83, SD 11.84 years) [25,79-83]. Because several reports draw on shared parent cohorts (eg, ICICLE-GAIT [Incidence of Cognitive Impairment in Cohorts with Longitudinal Evaluation-GAIT], V-TIME [Virtual Reality-Treadmill Combined Intervention for Enhancing Mobility and Reducing Falls in the Elderly], Mobilise-D, and FallRiskPD), these totals represent the sum of report-level sample sizes and include some individuals more than once, rather than counts of unique participants. The PRISMA flow diagram is shown in Figure 1. The remaining 28 reports were excluded because of a lack of gait quality parameter or real-world data or because they were non–peer-reviewed articles. Not all included reports contributed data to the meta-analysis, as reported gait parameters varied widely. After excluding duplicate datasets, 21 unique datasets were available for the meta-analysis in PD. The evidence base differed substantially between the 2 populations, with 37 PD reports compared with 6 stroke reports, several of which relied on overlapping datasets. All stroke-related findings should therefore be interpreted with caution and considered preliminary throughout this review. Study characteristics are summarized in Table 1.

**Table 1.** Characteristics of the included reports^a^.

Reports	Sample, n	Clinical concept of interest	Gait assessment	Monitoring period	Digital outcome	Algorithm validation	Sensors, n
Parkinson							
Weiss et al (2014)^b^ [70]	107	Fall risk	Lab^c^+RW^d^	3 days	Gait macro and micro	Not available	1
Weiss et al (2015)^b^ [71]	107	Cognitive function	Lab+RW	3 days	Gait macro and micro	Not available	1
Weiss et al (2015)^b^ [73]	72	Gait	Lab+RW	3 days	Gait macro and micro	Not available	1
Toosizadeh et al (2015) [72]	15	Gait	Lab+RW	1 day	Gait speed	Indoor in prior study [84]	1
Del Din et al (2016)^e^ [67]	47	Gait	Lab+RW	7 days	Gait macro and micro	Indoor in prior study [85]	1
Mancini et al (2018) [69]	24	Freezing of gait	RW	7 days	Gait macro and micro	Not available	3
Del Din et al (2019)^e,f^ [44]	170	Fall risk	Lab+RW	7 days	Gait macro and micro	Indoor in prior study [85]	1
Galperin et al (2019)^f^ [68]	125	Gait, physical activity	Lab+RW	7 days	Gait macro and micro	Not available	1
Del Din et al (2020)^f^ [43]	128	Fall risk	Lab+RW	7 days	Gait macro	Indoor in prior study [85]	1
Terashi et al (2020) [54]	106	Cognitive function, motor abnormalities	RW	1 day	Gait micro	Not available	1
Coates et al (2020)^e^ [53]	5	Gait	RW	7 days	Sample entropy of stride time	Indoor in prior study [85]	1
Shah et al (2020) [55]	16	Gait	Lab+RW	7 days	Gait macro and micro	Indoor in prior study [86]	3
Shah et al (2020)^g^ [61]	29	Gait	RW	7 days	Gait macro and micro	Indoor in prior study [86]	3
Shah et al (2020)^g^ [62]	29	Gait	RW	7 days	Gait macro and micro	Indoor in prior study [86]	3
Bouça-Machado et al (2021) [56]	24	Gait	Lab+RW	3 days	Gait macro and micro	Indoor in prior study [85]	1
Ullrich et al (2021)^h^ [57]	12	Gait tests	RW	14 days	Gait micro	Indoor in prior study [87]	2
Adams et al (2021) [49]	17	Gait, physical activity, tremor	Lab+RW	2 days	Gait macro and micro	Not available	5
Atrsaei et al (2021)^i^ [48]	27	Gait	Lab+RW	1 day	Gait speed	Indoor in prior study [88]	1
Atrsaei et al (2021) [74]	26	Fear of falling	Lab+RW	14 days	Gait macro and gait speed	Indoor in prior study [88]	1
Corrà et al (2021)^i^ [63]	27	Gait speed	Lab+RW	1 day	Gait speed	Indoor in prior study [88]	2
Rehman et al (2022)^e^ [50]	47	Parkinson disease classification	Lab+RW	7 days	Gait macro and micro	Indoor in prior study [85]	1
Roth et al (2022)^h^ [51]	38	Stairs, fall risk	RW	14 days	Gait micro and stairs	Outdoor in prior study [89]	2
Ullrich et al (2023)^h^ [58]	35	Fall risk	RW	14 days	Gait micro	Indoor in prior study [87]	2
Caballol et al (2023) [59]	39	Gait, freezing of gait, dyskinesia	RW	7 days	Gait macro and micro	Not available	1
Cohen et al (2023) [60]	46	Gait	Lab+RW	7 days	Gait macro and micro	Not available	1
Salis et al (2023)^j^ [24]	20	Gait	Lab+RW	2.5 hours	Gait macro and micro	Indoor within study	7
Micó-Amigo et al (2023)^j^ [52]	20	Gait	RW	2.5 hours	Gait macro and micro, gait events	Outdoor within study	1
Kirk et al (2023)^e^ [28]	62	Gait	RW	7 days	Gait speed	Indoor in prior study [85]	1
Romijnders et al (2023)^j^ [26]	18	Gait	RW	2.5 hours	Gait micro, gait events	Outdoor within study	2
Shah et al (2023) [75]	34	Fall risk	Lab+RW	7 days	Gait micro	Indoor in prior study [86]	3
Mirelman et al (2024)^f^ [64]	587	Gait	RW	7 days	Gait macro and micro	Indoor in prior study [85]	1
Kirk et al (2024)^j^ [27]	15	Gait speed	Lab+RW	2.5 hours	Gait speed	Indoor and outdoor within study	1
Zampogna et al (2024) [65]	48	Freezing of gait, dyskinesia	RW	6‐8 days	Gait micro, freezing, dyskinesia	Not available	1
Nishi et al (2024) [66]	41	Gait speed	Lab+RW	7 days	Gait micro	Indoor in prior study [85]	1
Kirk et al (2026) [76]	39	Contextualization	Lab+RW	7 days	Gait macro and micro	Indoor and outdoor in prior study [27,52]	1
Sidoroff et al (2026) [77]	41	Gait	Lab+RW	7 days	Gait macro and micro	Outdoor in prior study [90]	2
Yarnall et al (2026) [78]	531	Gait	Lab+RW	7 days	Gait macro and micro	Indoor and outdoor in prior study [27,52]	1
Stroke							
Sanchez et al (2015) [79]	23	Gait recovery	RW	3 ×1 days	Gait macro and micro	Not available	3
Punt et al (2016)^k^ [80]	56	Fall risk	RW	7 days	Gait macro and micro	Indoor in prior study [91]	1
Moore et al (2017) [25]	25	Gait	Lab+RW	2×7 days	Gait macro and micro	Indoor in prior study [85]	1
Punt et al (2017)^k^ [81]	40	Fall risk	Lab+RW	7 days	Gait macro and micro	Indoor within study	1
van de Port et al (2020)^k^ [82]	38	Gait	Lab+RW	7 days	Gait macro and micro	Indoor in prior study [91]	1
Felius et al (2025) [83]	35	Gait	Lab+RW	2 days	Gait speed, number of strides	Not available	1

a For the meta-analysis, only data from the real-world setting were used.

b Data originates from the same study population [92].

c Lab: gait was assessed in a laboratory or clinical setting.

d RW: gait was assessed in a real-world setting.

e Data originates from the ICICLE-GAIT (Incidence of Cognitive Impairment in Cohorts with Longitudinal Evaluation-GAIT) study (June 2009-December 2011).

f Data originates from the V-TIME (Virtual Reality-Treadmill Combined Intervention for Enhancing Mobility and Reducing Falls in the Elderly) study (November 2012-April 2015).

g Data originates from the same study population.

h Data originates from the FallRiskPD study (March 2019-June 2021).

i Data originates from the same study population.

j Data originates from the Mobilise-D study (July 2020-September 2021).

k Data originates from the same study population.

Research activity in this field has increased markedly, with more than 70% of included reports published in 2020 or later [24,26-28,43,48-66,74-78,82,83]. The United Kingdom contributed the largest number of reports (n=15), including 9 from multicenter collaborations [24,26,27,43,44,52,64,68,78]. Among the included reports, 18 PD reports [24,26-28,43,44,49,50,52,53,55,61,62,64,67,72,76,78] and 2 stroke reports [79,80] also included a comparison group of older adults. In total, 9 reports provided open-access datasets [24,26,27,49,51,52,57,58,78], 17 reported data availability upon request [25,28,48,50,54-56,59,60,62-65,74,76,77,83], and the remaining reports did not address data availability [43,44,53,61,66-73,75,79-82]. Algorithm transparency was limited overall. A total of 5 reports made their algorithms publicly available [26,52,64,76,78], and 1 offered access upon request [48].

### Methodological Quality

Methodological quality was evaluated across the 3 retained domains: reporting, external validity, and internal validity. Ratings for individual reports are provided in Multimedia Appendix 2. Overall quality scores in PD ranged from 69.4% to 94.4%, with a mean of 83.5% (SD 5.8%). Overall quality scores in stroke ranged from 72.2% to 91.7%, with a mean of 81.9% (SD 7.2%). Of the 37 PD reports, 32 were rated as high quality (scores above 75%), and 5 as moderate (scores between 50.1% and 75%). Of the 6 stroke reports, 4 were rated as high quality, and 2 as moderate. Reporting quality was high in both PD (mean 94.1%, SD 5.9%) and stroke (mean 93.3%, SD 6.8%). The main reporting deficiencies across both populations concerned protocol descriptions, walking bout and step detection definitions, and the characterization of participants. In stroke, an additional reporting gap was identified in the presentation of statistical results, with several reports presenting significance thresholds rather than exact *P* values for their main outcomes. External validity scored lowest across both populations, with mean scores of 49.3% (SD 15.9%) in PD and 45.8% (SD 18.8%) in stroke. The primary issues were failure to confirm participant representativeness, absence of participation bias assessment, and lack of algorithm validation within the target population. Internal validity scores were substantially higher in both PD (mean 91.2%, SD 9.3%) and stroke (mean 89.6%, SD 5.1%), with the main concern being that some reports did not fully address the appropriateness of statistical methods. Across both populations, external validity represented the most consistently weak domain.

### Methodological Protocols

The included reports addressed a broad range of research objectives, reflecting the heterogeneity of the field. In PD, study designs ranged from technical algorithm validation to clinical characterization and intervention evaluation, while stroke reports were more limited in number and scope. Of the 37 PD reports, 23 assessed gait in both laboratory and real-world settings [24,27,43,44,48-50,55,56,60,63,66-68,70-78], while 14 reports focused exclusively on real-world [26,28,51-54,57-59,61,62,64,65,69]. Disease severity was characterized using the Unified Parkinson’s Disease Rating Scale across all PD reports [24,26-28,43,44,48-78], providing a consistent clinical reference point. Four reports focused on technical validation, examining the accuracy of gait detection algorithms in laboratory or real-world contexts [24,26,27,52]. Construct validity was assessed in 20 reports through known-groups, convergent, and discriminant approaches. These included comparisons between people with PD and healthy controls, fallers and nonfallers, people with and without freezing of gait, and disease severity groups, as well as associations between home-based gait parameters, in-clinic assessments, clinical scales, and patient-reported outcomes [28,44,48,49,51,53-55,61-65,68-73,78].

Four reports examined methodological questions relevant to real-world gait assessment, including how environment, walking bout length, and data aggregation strategies influence gait parameters and disease classification or fall risk [50,58,66,67]. Additional methodological contributions included GPS-derived indoor-outdoor contextualization and the detection of unsupervised gait tests within continuous sensor data [57,76]. Beyond validation, wearable sensors were used to evaluate intervention effects and clinical determinants of mobility. Three reports assessed ambulatory outcomes in the context of rehabilitation, examining both efficacy and effectiveness [56,60] or effectiveness alone [43]. Two reports investigated treatment-related effects, including changes following treatment adjustments [59] and differences in gait and turning markers between off- and on-levodopa states across clinical and daily-life settings [75]. In addition, one report investigated the influence of fear of falling on mobility [74], and another compared mobility profiles between PD and atypical parkinsonian syndromes [77].

The 6 stroke reports were more limited in methodological scope. A total of 4 assessed gait in both laboratory and real-world settings [25,81-83], while 2 focused exclusively on real-world settings [79,80]. One report evaluated the feasibility, validity, and test-retest reliability of a single lower back accelerometer for measuring gait after stroke in both settings [25]. Construct validity was supported through comparisons between fall-prone and nonfall-prone individuals with stroke and associations between real-world gait parameters and clinical measures including balance, gait speed, and fear of falling [79,80,82]. Predictive validity was examined in 2 reports investigating whether laboratory-based and daily-life gait characteristics could predict prospective falls or community walking outcomes [81,83]. Notably, none of the stroke reports used the National Institutes of Health Stroke Scale (NIHSS) to characterize stroke severity [25,79-83], limiting the ability to assess the representativeness of the study populations and the generalizability of findings across severity levels.

### Sensor Configuration

Sensor configurations varied considerably across reports in terms of sensor type, number, and placement. In PD, all reports used accelerometers [24,26-28,43,44,48-78], with gyroscopes incorporated in 17 reports [24,26,48,51,52,55,57,58,61-63,69,74-78] and magnetometers in 7 [24,55,61,62,69,74,75]. The number of sensors per report ranged from 1 to 7, with a mean of 1.70 (SD 1.29). In stroke, all reports also used accelerometers, with configurations ranging from 1 to 3 sensors per report [25,79-83]. In PD, the most common configuration was a single sensor positioned on the lower back, used in 20 reports [27,28,43,44,50,52,53,56,60,64-68,70,71,73,74,76,78], secured with tape [28,43,44,53,60,64,67,68,76,78], a belt [27,52,65,70,71,73,74,78], both tape and a belt [78], or by a method that was not reported [50,56,66]. Less frequently, reports paired a lower back sensor with additional sensors on the feet [69] or ankles [55,61,62,75]. Other reports used sensors placed exclusively on one [48] or both feet [26,51,57,58,63,77], while more complex multisensor setups included the inertial module with distance sensors and pressure insoles system, comprising 3 IMUs on the lower back and both feet, distance sensors, and pressure insoles [24]. Additional configurations included sensors placed at the chest [54,72], the left hip [59], and a 5-sensor setup distributed across the thighs, forearms, and trunk [49]. In stroke, 4 reports used a single lower back sensor [25,80-82] secured with a belt [80-82] or tape [25]. One report used a single sensor attached to the right foot [83], and one report used 3 sensors placed on the chest and both thighs [79]. In PD, the most frequently used device was the AX3 triaxial accelerometer (Axivity), used in 12 reports [28,43,44,50,53,56,60,64,66-68,76], followed by the McRoberts DynaPort MM+ in 6 reports [27,52,70,71,73,78], the Opal sensor (APDM) in 5 reports [55,61,62,69,75], and the Mobile-GaitLab system (Portabiles Healthcare Technologies GmbH) in 4 reports [51,57,58,77]. In PD, 20 reports used a sampling frequency of 100 Hz [24,26-28,43,44,50,52-54,56,60,64,66-68,70,71,76,78]. In stroke, the most frequently used device was the McRoberts DynaPort MM+ in 3 reports [80-82]. Notably, all 3 of these reports were based on the same dataset, which substantially limits the independence of the stroke evidence base for device-specific observations. In total, 4 stroke reports used a sampling frequency of 100 Hz [25,80-82], 1 of 104 Hz [83], and 1 of 128 Hz [79].

### Technical Validation

Technical validation for real-world gait algorithms was reported in 27 of 37 PD reports [24,26-28,43,44,48,50-53,55-58,61-64,66,67,72,74-78]. For the remaining 10 reports, the technical validation status was unclear, as no comparison against an external reference system was reported or could be clearly traced from the cited methodology [49,54,59,60,65,68-71,73]. Of the 27 validated reports, 3 had been validated exclusively in healthy controls [57,58,72], a limitation when interpreting their applicability to clinical populations. Among the validated reports, 20 validations were conducted indoors [24,28,43,44,48,50,53,55-58,61-64,66,67,72,74,75], 4 outdoors [26,51,52,77], and 3 combined both settings [27,76,78]. Notably, 9 indoor reports [28,43,44,50,53,56,64,66,67] based their algorithms on the work of Del Din et al [85], using GAITRite as the reference system, which was the most commonly used reference overall [28,43,44,50,53,55-58,61,62,64,66,67,75]. Other validation approaches included stereophotogrammetry, the inertial module with distance sensors and pressure insoles system, force-sensing insoles, or video analysis, reflecting considerable variability in reference systems across the literature [24,26,27,48,51,52,63,72,74,76-78]. In total, 4 of the 6 stroke reports described algorithm validation [25,80-82], all conducted indoors using video analysis [80-82] or a combination of GAITRite and Opal sensors [25]. For the remaining 2 reports, the technical validation status was unclear [79,83].

### Observation Windows and IMU-Derived Outcomes

A 7-day (range 6‐8 days in 1 report) observation window was the most common monitoring duration for real-world gait assessment across both PD and stroke reports [25,28,43,44,50,53,55,59-62,64-69,75-78,80-82]. In PD, monitoring durations ranged from 2.5 hours to 14 days, though the shortest duration was used exclusively for algorithm validation rather than real-world gait assessment [24,26,27,52]. In stroke, monitoring durations ranged from 1 to 7 days [25,79-83]. Continuous walking sequences, referred to as walking bouts, were extracted from sensor recordings across the included reports. However, the criteria used to define a walking bout varied considerably. Overall, 13 distinct definitions were identified across the included reports (Table 2), with 10 in PD, 2 in stroke, and 1 in both cohorts. Some reports applied minimum step or stride count thresholds, others used minimum duration criteria, and some incorporated rest period rules to determine whether consecutive walking sequences should be merged or treated separately. Notably, 17 reports did not provide a walking bout definition [48,49,54,56,57,59,60,64,65,68,70-74,79,83].

**Table 2.** Walking bout definitions applied in included reports, presented separately for Parkinson disease and stroke^a^.

	References
Parkinson disease reports	
Minimum bout length of 3 steps with no threshold set for maximum resting period between consecutive walking bouts.	[50,53,66,67]
Minimum bout length of 5 strides with a maximum resting period of 3.5 seconds.	[51]
Minimum bout length of 3 steps with a maximum resting period of 2.5 seconds.	[28,44]
Minimum bout length of greater than 10 seconds with a maximum resting period of 2.5 seconds.	[43]
Minimum bout length of 3 steps and 3 seconds with a maximum resting period of 2.5 seconds.	[55,61,62,75]
Minimum bout length of 4 strides with a maximum resting period of 3 seconds.	[58]
Minimum of 2 consecutive strides of both feet with a maximum resting period of 3 seconds.	[24,26,27,52,78]
Minimum of 2 consecutive strides of both feet with a resting period or any other activity (nonwalking period).	[76]
Minimum bout length of 3 consecutive strides and 3 seconds with a maximum resting period of 6 seconds.	[69]
Consecutive gait cycles less than 3 seconds apart.	[63]
A sequence of consecutive steps.	[77]
Stroke reports	
Minimum bout length of 3 steps with no threshold set for maximum resting period between consecutive walking bouts.	[25]
An episode of 1 or more consecutive gait epochs of 8 seconds.	[82]
Gait activity of a minimum of 8 seconds.	[80,81]

a The table summarizes the operational criteria used to define walking bouts, including minimum step or stride count thresholds, minimum duration criteria, and interruption rules. A total of 13 distinct definitions were identified across the included literature. In total, 17 reports did not provide a walking bout definition and are therefore not represented in this table.

**Figure 2.**
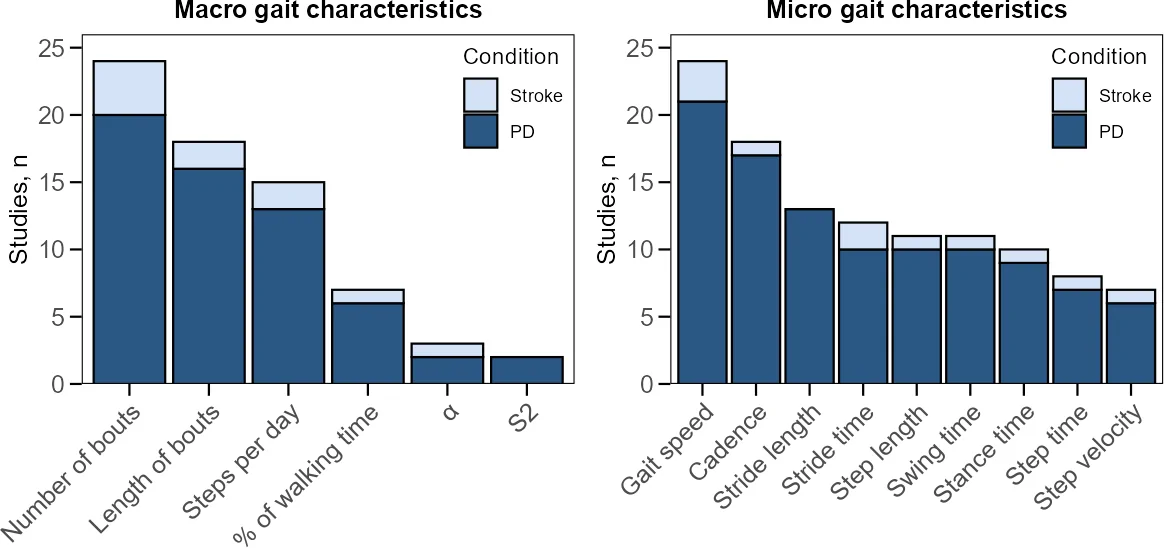
Frequency of macro and micro gait characteristics in real-world gait assessment across included reports for Parkinson disease (n=37) and stroke (n=6).

The digital outcomes extracted in the included reports were categorized using the framework of macro and micro gait characteristics [42]. Figure 2 summarizes the frequency with which individual parameters were reported across PD and stroke reports. Across both populations, the most commonly reported macro characteristics were the number and length of walking bouts and the number of steps per day. Micro characteristics showed greater variability in reporting across both populations. In PD, the most frequently reported micro characteristics were gait speed, cadence, and stride length, while in stroke, they were gait speed and stride time. Beyond these, a range of additional micro parameters were reported across the literature, though each appeared in too few reports to allow meaningful cross-study comparisons, reflecting the considerable heterogeneity in outcome reporting across the included reports.

### Meta-Analysis

**Figure 3.**
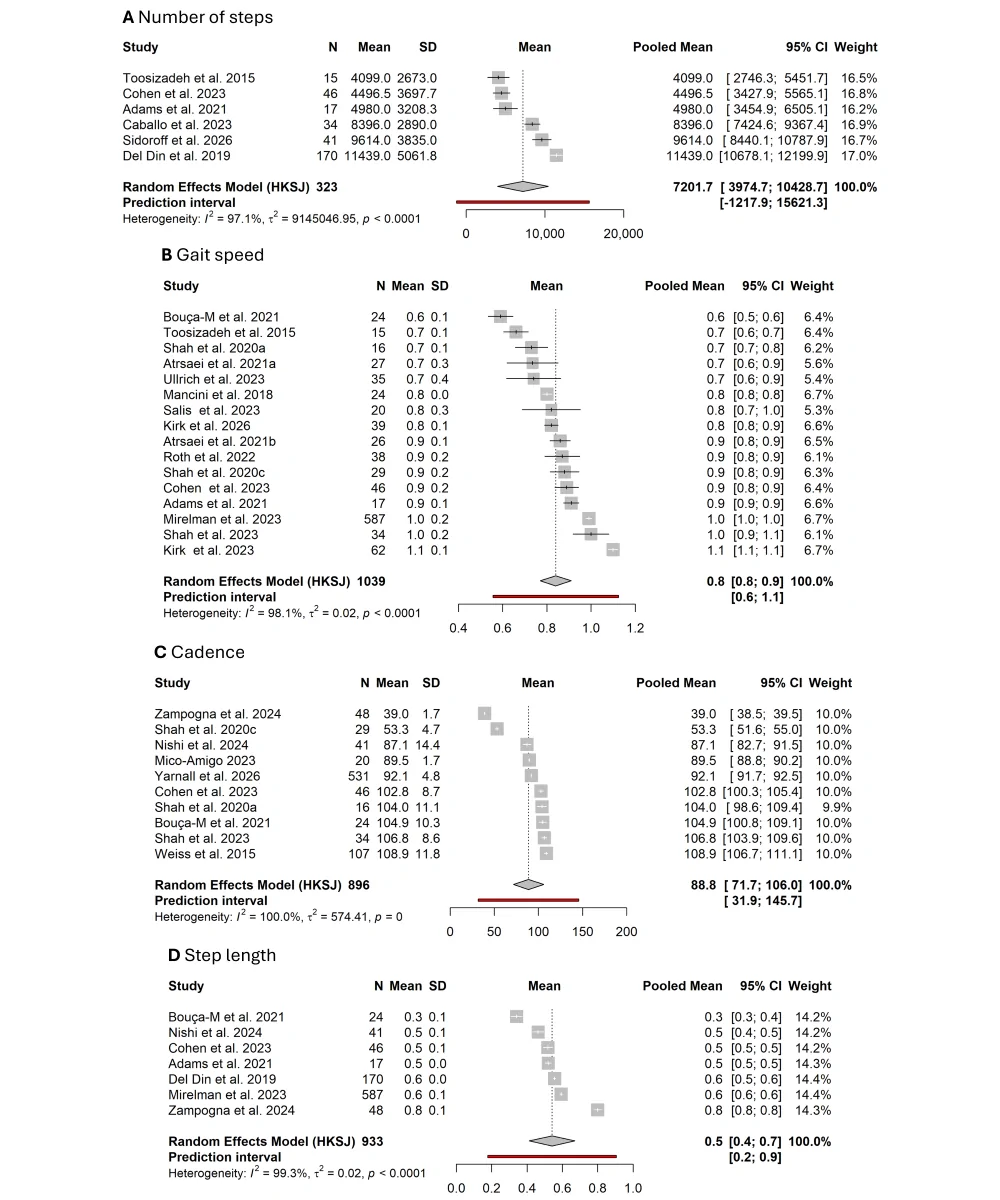
Random-effects models for Parkinson disease macro (A) number of steps per day and micro (B) gait speed, (C) cadence, and (D) step length gait parameters measured during real-world gait assessment. The dashed line indicates the pooled mean estimate, while the diamond shape represents the 95% CIs [24,28,44,48,49,51,52,55,56,58-60,62,64-66,69,71,72,74-78]. HKSJ: Hartung-Knapp-Sidik-Jonkman.

Because of the limited number of compatible stroke reports, the quantitative synthesis was conducted exclusively on the PD cohorts. Four real-world gait parameters met the criteria for meta-analysis: number of steps per day (k=6), gait speed (k=16), cadence (k=10), and step length (k=7). Between-study heterogeneity was extreme for all parameters: in every model, the 95% PI was substantially wider than the CI. The pooled mean for the number of steps per day was 7202 (95% CI 3975-10,429). Notably, the PIs for this macro parameter extended below zero, a mathematical impossibility that quantitatively highlights the profound variance introduced by differing aggregation rules and monitoring durations across reports. For gait speed, the pooled mean was 0.84 m/s (95% CI 0.77-0.91). However, the 95% PI spanned from 0.56 to 1.12 m/s, indicating that a future real-world assessment could reasonably fall anywhere within this wide range. Similarly, cadence yielded a pooled mean of 88.81 steps per minute (95% CI 71.65-105.92; 95% PI 31.94-145.68), and step length was 0.54 m (95% CI 0.41-0.67; 95% PI 0.18-0.91). Forest plots detailing the Hartung-Knapp-Sidik-Jonkman models and PIs for the most frequently reported macro and micro parameters are provided in Figure 3.

While a meta-analysis was not possible for stroke because of the limited number of reports, 4 reports provided the number of bouts, with means of 126.1 (SD 69.8) [80], 104.83 (SD 61.16) [79], 123.3 (SD 61.3) [82], and 535 (SD 213.49) [25] bouts. Bout length was reported in 2 reports, with means of 17.1 (SD 2.6) and 16.46 (SD 11.27) seconds [79,80]. In total, 2 reports provided step counts, with means of 7825 (SD 6428) and 3048 (SD 1983) steps [25,82]. Gait speed was provided in 3 reports (mean 0.45, SD 0.13 m/s [83]; mean 0.71, SD 0.15 m/s [80]; mean 0.69, SD 0.15 m/s [81]), while stride time was reported in 2 (mean 1.34, SD 0.32 seconds and mean 1.37, SD 0.37 seconds) [80,81]. Step length (mean 0.61, SD 0.09 m) [25], step velocity (mean 1.05, SD 0.17 m/s) [25], cadence (mean 90.3, SD 13.8 steps per minute) [82], swing time (mean 0.47, SD 0.03 seconds) [25], and stance time (mean 0.76, SD 0.04 seconds) [25] were each available from a single report.

### Certainty of Evidence

Certainty of evidence was very low for all 4 meta-analyzed gait outcomes (Table 3), driven primarily by very serious inconsistency, evidenced by PIs that far exceeded the corresponding CIs. Notably, risk of bias was not serious for any outcome, as no contributing report scored below 75% on the modified quality score. The very low certainty therefore reflects methodological fragmentation across reports rather than deficiencies within them. The 2-level downgrade for inconsistency alone was sufficient to reach the lowest possible certainty rating, making the overall judgment robust regardless of the ratings assigned to the remaining domains.

**Table 3.** GRADE^a^ certainty of evidence for meta-analyzed real-world gait outcomes in Parkinson disease.

Pooled mean (95% CI)	Studies, n	Risk of bias	Inconsistency	Indirectness	Imprecision	Publication bias	Overall certainty of evidence
Number of steps per day							
7202 (3975-10,429)	6	Not serious	Very serious^b^	Serious^c^	Serious^d^	Not assessed^e^	⨁◯◯◯ Very low^b,c,d^
Gait speed (m/s)							
0.84 (0.77‐0.91)	16	Not serious	Very serious^b^	Serious^c^	Not serious	Not assessed^e^	⨁◯◯◯ Very low^b,c^
Cadence (steps per minute)							
88.81 (71.65‐105.92)	10	Not serious	Very serious^b^	Serious^c^	Serious^d^	Not assessed^e^	⨁◯◯◯ Very low^b,c,d^
Step length (m)							
0.54 (0.41‐0.67)	7	Not serious	Very serious^b^	Serious^c^	Serious^d^	Not assessed^e^	⨁◯◯◯ Very low^b,c,d^

a GRADE: Grading of Recommendations Assessment, Development, and Evaluation.

b Downgraded 2 levels for extreme between-study heterogeneity, indicated by PIs far wider than the CIs.

c Downgraded 1 level because external validity was the weakest quality domain, reflecting limited evidence of participant representativeness, and gait-detection algorithms validated predominantly indoors, in prior studies, or outside the target population.

d Downgraded 1 level because the 95% CI around the pooled mean was wide (relative width ≥30% of the pooled estimate).

e Funnel-plot asymmetry testing was not performed for any outcome. For 2 outcomes, steps per day (k=6) and step length (k=7), fewer than 10 studies were available, which precludes reliable testing. For the remaining 2 outcomes, gait speed (k=16) and cadence (k=10), heterogeneity was too extreme, as reflected in very wide PIs, making any asymmetry test uninterpretable even with an adequate number of studies. No downgrade was applied, although the absence of a gray-literature search and the restriction to English-language publications remain potential unassessed sources of bias.

## Discussion

### Principal Findings

This systematic review and meta-analysis provides a comprehensive synthesis of real-world gait assessment using wearable sensors in PD and stroke, examining sensor configurations, validation strategies, outcome reporting practices, and methodological approaches across the available literature. The findings reveal a field that is technically advancing but methodologically fragmented. In PD, a substantial and growing body of research supports the use of IMU-based wearable sensors for real-world gait assessment, with most reports demonstrating high methodological quality and broad coverage of both technical and construct validation. However, considerable heterogeneity was identified across sensor configurations, walking bout definitions, outcome reporting practices, and validation approaches, which the meta-analysis confirmed through consistently wide PIs and high between-study variance. Although overall methodological quality was high, external validity emerged as the weakest domain across both populations, driven by insufficient participant representativeness and limited algorithm validation within the target population, a finding that directly constrains the generalizability of current real-world gait assessment research.

In stroke, the evidence base remains critically underdeveloped, with a small number of reports, several of which drew on overlapping datasets, precluding meaningful quantitative synthesis; therefore, all stroke-related findings in this review must be interpreted as preliminary. This disparity is particularly striking, given the global burden of stroke [3]. Unlike PD research, which has benefited from coordinated multicenter efforts, poststroke monitoring has developed in a fragmented and isolated manner. The substantial methodological groundwork laid by initiatives such as Mobilise-D, including validated sensor protocols, harmonized digital mobility outcomes, and shared algorithmic frameworks, represents a significant opportunity for stroke rehabilitation [93]. Rather than building from scratch, these established frameworks could be critically appraised and adapted for poststroke populations, providing a strong and efficient foundation for developing a robust, independent evidence base for real-world gait monitoring after stroke.

### Sensor Configuration and Placement Considerations

Selecting an optimal sensor configuration for real-world gait assessment requires balancing measurement accuracy, clinical usability, patient acceptability, and practical feasibility [94], and the considerable variation in sensor setups observed across the included reports reflects exactly these competing demands. A single sensor positioned at the lower back was the most commonly used configuration in PD and stroke, consistent with broader trends in the field [95]. Placed near the body’s center of mass, the lower back sensor captures whole-body movement patterns, is straightforward to attach, and is generally well tolerated during prolonged monitoring [96,97]. However, trunk-mounted sensors present known challenges in detecting gait events such as initial and final foot contacts, which can affect spatiotemporal accuracy and drift correction [98]. Given that the lower back reflects whole-body movement rather than individual limb kinematics, sensors placed closer to the point of ground contact, such as at the shank or foot, may offer greater accuracy in detecting step-to-step and side-to-side differences that are clinically relevant in this population [99,100]. Whether this translates into meaningfully improved clinical sensitivity remains an open question, as no report in this review directly compared sensor placements in stroke populations. It should also be noted that estimating asymmetry from 2 independently placed sensors introduces the risk of misalignment and calibration errors, a technical challenge that future work in this area will need to carefully account for [101,102]. Beyond placement, the method of sensor attachment influences usability and compliance during prolonged monitoring [103]. Adhesive attachment may reduce patient burden and promote consistent sensor placement during prolonged monitoring, whereas shoe-mounted sensors, although precise, may be constrained by footwear type, sensor fixation, orthopedic considerations, or settings in which people commonly remove shoes indoors [104,105]. Sensor configuration choices should therefore be guided by the target population’s clinical profile, practical context, and the specific gait features of interest [98].

### Technical Validation Protocols and Ecological Validity

Despite the well-recognized complexity of real-world walking conditions [24], most validations in the included reports relied on simplified protocols involving uniform walking tasks that do not reflect the dynamic nature of daily life. In response, the most recent studies have introduced multitask and multicontext validation protocols simulating real-world factors such as speed variations, inclines or steps, surface types, path shapes, and cognitive load [106,107]. While these elaborated scenarios provide a more comprehensive assessment of algorithm performance, they are still conducted in controlled settings [106,107]. Ecological validation in outdoor, less standardized environments remains the exception, primarily because of the logistical demands of benchmarking sensor-derived data in natural settings [24]. As the field progresses, advancing and standardizing validation methodologies will be essential to ensure that algorithms are rigorously tested under realistic conditions.

### Walking Bout Definitions and Observation Windows

The 13 distinct walking bout definitions identified in this review illustrate a challenge that has been recognized more broadly in the field, as the criteria used to define walking sequences directly determine which data are analyzed and which are excluded, affecting both macro and micro gait parameters [67,108]. Kluge et al [109] used a Delphi consensus process to establish a common terminological framework for real-world walking, defining a walking bout as a sequence of at least 2 consecutive strides of both feet, with start and end determined by a resting period or any other nonwalking activity. The substantial proportion of reports in this review that did not report their walking bout definition suggests that this framework has not yet been widely adopted. Standardization of walking bout criteria is not merely a methodological convenience; it is a prerequisite for meaningful cross-study comparison and the development of reproducible gait benchmarks [110,111].

The observation window over which data are collected represents an equally important consideration. A 7-day monitoring period was the most common duration across both populations in this review, a choice supported by recent evidence from Buekers et al [112], who demonstrated that minimum wear time and number of valid days required for reliable weekly digital mobility outcomes are digital mobility outcome–specific and consistent across health conditions. By expert consensus, more than 12 hours of daily wear time across at least 3 valid days is recommended for studies involving multiple outcomes or conditions, though specific parameters may require more days [112]. Despite this, protocols should still target 7 consecutive days to account for participant adherence and technical issues [112].

### Sources of Heterogeneity and Certainty of Evidence

The extreme heterogeneity observed in the meta-analysis, evident in PIs far wider than the CIs, is perhaps the most clinically important finding of this review, formally reflected in very low certainty of evidence ratings across all 4 outcomes, driven by very serious inconsistency and serious indirectness throughout. The wide PIs and high between-study variance confirm that pooled point estimates cannot be interpreted as definitive clinical benchmarks for real-world gait in PD. Rather, they serve as quantitative evidence of the substantial methodological fragmentation in the field. Critically, this uncertainty does not reflect poor quality within individual reports, as reporting and internal validity were consistently strong, but rather the absence of methodological consensus across them. Major procedural inconsistencies concerned hardware placement, walking bout definitions, data aggregation protocols, and outcome nomenclature. These were compounded by limited participant representativeness, and algorithm validation predominantly conducted in simplified laboratory conditions rather than in ecologically valid real-world settings, preventing meaningful comparability. Even when pooling was restricted to reports with comparable bout definitions, considerable residual variance remained. Because inconsistent reporting of clinical covariates (eg, disease severity stages) precluded meta-regression, the exact proportion of variance attributable to clinical versus procedural factors could not be partitioned. A further limitation was the absence of detailed contextual information regarding assistive device use and the severity of gait impairments, both of which substantially influence real-world gait patterns and algorithm performance, further constraining the comparability and generalizability of the findings [52,113]. These findings carry a clear implication, as certainty in real-world gait estimates will not improve through additional studies of the current design alone, and it is therefore imperative that future research, particularly in emerging fields such as poststroke real-world monitoring, adopts harmonized approaches early to avoid compounding the methodological fragmentation currently observed in the PD literature.

### The Role of Context in Real-World Gait Assessment

The purpose and context of walking itself remain underexplored in current real-world gait research. In daily life, walking serves diverse functional goals such as navigating the home, community ambulation, exercise, or social participation, each of which may elicit different gait patterns and place different demands on the individual [106,114]. Gait metrics derived from short indoor walking sequences are therefore not directly comparable to those captured during longer community outings, as gait characteristics vary by environment and ambulatory bout length [67]. However, most analyses did not explicitly stratify outcomes by these contextual factors. Kirk et al [76] demonstrated this empirically in PD, showing that integrating GPS-derived indoor and outdoor contextual information with wearable-derived gait outcomes revealed meaningful differences in walking behavior across environments, with between-group differences emerging only during outdoor walking. Without contextual awareness, clinically relevant differences in gait may therefore be obscured or misattributed. Beyond environmental context, internal biological and psychological factors including sleep quality, nutritional state, hormonal fluctuations, and anxiety may introduce within-person variability that is difficult to separate from disease-related fluctuations; yet, these are not accounted for in current real-world monitoring studies [115-118].

### Limitations

This review has several limitations that should be considered when interpreting its findings. First, only studies published in English were included, which may have introduced language bias and may have led to the exclusion of relevant evidence published in other languages. Second, multiple included reports were derived from overlapping datasets, which may have introduced bias toward specific methodological approaches and reduced the diversity of the evidence base. This concern is particularly acute in stroke, where the small number of reports and their limited independence substantially constrain the conclusions that can be drawn. Third, the review focused exclusively on macro and micro gait characteristics, and important aspects of real-world mobility such as turning, freezing of gait, and postural transitions were not examined. Finally, despite strict data extraction criteria applied prior to pooling, residual heterogeneity limited the clinical utility of the pooled estimates and precluded meaningful subanalyses by clinical variables such as disease severity, faller status, or motor fluctuation state. Although the small number of reports providing compatible metrics constrained these analyses, this limitation reflects the emerging nature of the field rather than a flaw in the review methodology and should serve as a clear signal of where standardization efforts are most urgently needed.

### Conclusions

Unlike previous reviews that have focused narrowly on single diseases, specific hardware, or classification algorithms, this review is novel in contrasting PD and stroke populations to highlight shared assessment challenges. It demonstrates that wearable sensor-based real-world gait assessment in PD is technically advancing but methodologically fragmented, while the stroke evidence base remains critically underdeveloped. By quantifying this heterogeneity, this review brings to the field a critical recognition: the observed variance is substantially compounded by procedural and algorithmic inconsistencies, limiting the ability to isolate genuine clinical differences. Important foundational work has already been undertaken to address this, including a comprehensive roadmap for integrating wearable sensors into clinical practice and a Delphi consensus establishing a common terminological framework for real-world walking [5,109]. In stroke, the sparse evidence base represents an opportunity to critically assess and adopt these harmonized frameworks early. Resolving these methodological inconsistencies carries profound real-world implications. Collaborative standardization of terminology, validation procedures, and core outcomes is essential to transition wearable sensors from exploratory research tools to reliable clinical instruments. Ultimately, establishing reliable digital mobility outcomes will support the International Classification of Functioning, Disability and Health’s underused construct of performance, enabling personalized care, precise remote monitoring, and an improved picture of functional mobility in everyday life.

## Supplementary material

10.2196/93413 Multimedia Appendix 1 Search strategy per database.

10.2196/93413 Multimedia Appendix 2 Quality checklist, calculation of quality score, and quality score per study.

10.2196/93413 Checklist 1 PRISMA checklist.

10.2196/93413 Checklist 2 PRISMA-S checklist.

10.2196/93413 Checklist 3 PRISMA 2020 Abstract checklist.

## Abbreviations

CI: confidence interval
GRADE: Grading of Recommendations Assessment, Development, and Evaluation
ICICLE-GAIT: Incidence of Cognitive Impairment in Cohorts with Longitudinal Evaluation-GAIT
IMU: inertial measurement unit
PD: Parkinson disease
PI: prediction interval
PRISMA: Preferred Reporting Items for Systematic Reviews and Meta-Analyses
PRISMA-S: Preferred Reporting Items for Systematic Reviews and Meta-Analyses Literature Search Extension
V-TIME: Virtual Reality-Treadmill Combined Intervention for Enhancing Mobility and Reducing Falls in the Elderly
