# Supplementary material for: Wearable Sensors in Gait Assessment for Parkinson Disease and Stroke in Real-World Environments: Systematic Review and Meta-Analysis
Source: J Med Internet Res. 2026 Sep 25;28:e93413. doi: 10.2196/93413 (PMC13613968; doi:10.2196/93413)
Supplement: Supplementary file 1 [file jmir-v28-e93413-s001.docx]

| **PUBMED** |  | *("Parkinson Disease"[MeSH Terms] OR "Parkinsonian Disorders"[MeSH Terms] OR "Stroke"[MeSH Terms] OR "Ischemic Stroke"[MeSH Terms] OR "Hemorrhagic Stroke"[MeSH Terms] OR parkinson*[Title/Abstract] OR parkinsonian[Title/Abstract] OR parkinsonism[Title/Abstract] OR "Lewy body parkinson*"[Title/Abstract] OR PD[Title/Abstract] OR stroke[Title/Abstract] OR strokes[Title/Abstract] OR poststroke[Title/Abstract] OR "post-stroke"[Title/Abstract] OR "post stroke"[Title/Abstract] OR "cerebrovascular accident*"[Title/Abstract] OR "brain infarction"[Title/Abstract] OR "cerebral infarction"[Title/Abstract])* |
| --- | --- | --- |
|  | AND | *("Gait"[MeSH Terms] OR "Gait Analysis"[MeSH Terms] OR gait[Title/Abstract] OR walk*[Title/Abstract] OR ambulation[Title/Abstract] OR ambulat*[Title/Abstract] OR locomot*[Title/Abstract] OR stride*[Title/Abstract] OR "walking speed"[Title/Abstract] OR "gait speed"[Title/Abstract] OR "step length"[Title/Abstract] OR "step time"[Title/Abstract] OR "stride length"[Title/Abstract] OR "stride time"[Title/Abstract] OR "spatiotemporal"[Title/Abstract] OR "gait parameter*"[Title/Abstract] OR "gait characteristic*"[Title/Abstract])* |
|  | AND | *("Wearable Electronic Devices"[MeSH Terms] OR "Accelerometry"[MeSH Terms] OR "wearable electronic device*"[Title/Abstract] OR "wearable device*"[Title/Abstract] OR "wearable technolog*"[Title/Abstract] OR "wearable sensor*"[Title/Abstract] OR "wearable system*"[Title/Abstract] OR "body-worn sensor*"[Title/Abstract] OR "body-worn device*"[Title/Abstract] OR "body worn sensor*"[Title/Abstract] OR "body worn device*"[Title/Abstract] OR "inertial sensor*"[Title/Abstract] OR "inertial measurement unit*"[Title/Abstract] OR IMU[Title/Abstract] OR IMUs[Title/Abstract] OR acceleromet*[Title/Abstract] OR gyroscop*[Title/Abstract] OR magnetomet*[Title/Abstract] OR "motion sensor*"[Title/Abstract])* |
|  | AND | *("Monitoring, Ambulatory"[MeSH Terms] OR "daily life"[Title/Abstract] OR "daily living"[Title/Abstract] OR "everyday life"[Title/Abstract] OR "usual environment"[Title/Abstract] OR "real world"[Title/Abstract] OR real-world[Title/Abstract] OR "free living"[Title/Abstract] OR free-living[Title/Abstract] OR unsupervised[Title/Abstract] OR "remote monitoring"[Title/Abstract] OR "ambulatory monitoring"[Title/Abstract] OR "home environment"[Title/Abstract] OR "community setting*"[Title/Abstract] OR "community-dwelling"[Title/Abstract] OR "habitual environment"[Title/Abstract])* |
|  | NOT | *(Review[Publication Type] OR Systematic Review[Publication Type] OR Meta-Analysis[Publication Type] OR Editorial[Publication Type] OR Letter[Publication Type] OR Comment[Publication Type])* |
| **EMBASE** |  | *('parkinson disease'/exp OR 'parkinsonism'/exp OR 'stroke'/exp OR parkinson*:ti,ab OR parkinsonian:ti,ab OR parkinsonism:ti,ab OR 'lewy body parkinson*':ti,ab OR pd:ti,ab OR stroke:ti,ab OR strokes:ti,ab OR poststroke:ti,ab OR 'post-stroke':ti,ab OR 'post stroke':ti,ab OR 'cerebrovascular accident*':ti,ab OR 'brain infarction':ti,ab OR 'cerebral infarction':ti,ab)* |
|  | AND | *('gait'/exp OR 'gait analysis'/exp OR gait:ti,ab OR walk*:ti,ab OR ambulation:ti,ab OR ambulat*:ti,ab OR locomot*:ti,ab OR stride*:ti,ab OR 'walking speed':ti,ab OR 'gait speed':ti,ab OR 'step length':ti,ab OR 'step time':ti,ab OR 'stride length':ti,ab OR 'stride time':ti,ab OR spatiotemporal:ti,ab OR 'gait parameter*':ti,ab OR 'gait characteristic*':ti,ab)* |
|  | AND | *('wearable electronic device'/exp OR 'accelerometer'/exp OR wearable*:ti,ab OR 'wearable electronic device*':ti,ab OR 'wearable device*':ti,ab OR 'wearable technolog*':ti,ab OR 'wearable sensor*':ti,ab OR 'wearable system*':ti,ab OR 'body-worn sensor*':ti,ab OR 'body-worn device*':ti,ab OR 'body worn sensor*':ti,ab OR 'body worn device*':ti,ab OR 'inertial sensor*':ti,ab OR 'inertial measurement unit*':ti,ab OR imu:ti,ab OR imus:ti,ab OR acceleromet*:ti,ab OR gyroscop*:ti,ab OR magnetomet*:ti,ab OR 'motion sensor*':ti,ab)* |
|  | AND | *('ambulatory monitoring'/exp OR 'daily life':ti,ab OR 'daily living':ti,ab OR 'everyday life':ti,ab OR 'usual environment':ti,ab OR 'real world':ti,ab OR 'real-world':ti,ab OR 'free living':ti,ab OR 'free-living':ti,ab OR unsupervised:ti,ab OR 'remote monitoring':ti,ab OR 'ambulatory monitoring':ti,ab OR 'home environment':ti,ab OR 'community setting*':ti,ab OR 'community-dwelling':ti,ab OR 'habitual environment':ti,ab)* |
|  | NOT | *(review:it OR 'short survey':it OR 'conference abstract':it OR 'conference review':it OR editorial:it OR letter:it OR note:it)* |
| **Scopus** |  | *(TITLE-ABS-KEY ("Parkinson disease" OR "Parkinsonian disorder*" OR parkinson* OR parkinsonian OR parkinsonism OR "Lewy body parkinson*" OR PD OR stroke OR strokes OR poststroke OR "post-stroke" OR "post stroke" OR "cerebrovascular accident*" OR "brain infarction" OR "cerebral infarction" ))* |
|  | AND | *(TITLE-ABS-KEY(gait OR walk* OR ambulation OR ambulat* OR locomot* OR stride* OR "walking speed" OR "gait speed" OR "step length" OR "step time" OR "stride length" OR "stride time" OR spatiotemporal OR "gait parameter*" OR "gait characteristic*" ))* |
|  | AND | *(TITLE-ABS-KEY ("wearable electronic device*" OR "wearable device*" OR “wearable technolog*" OR "wearable sensor*" OR "wearable system*" OR "body-worn sensor*" OR "body-worn device*" OR "body worn sensor*" OR "body worn device*" OR "inertial sensor*" OR "inertial measurement unit*" OR IMU OR IMUs OR acceleromet* OR gyroscop* OR magnetomet* OR "motion sensor*" ))* |
|  | AND | *(TITLE-ABS-KEY("daily life" OR "daily living" OR "everyday life" OR "usual environment" OR "real world" OR "real-world" OR "free living" OR "free-living" OR unsupervised OR "remote monitoring" OR "ambulatory monitoring" OR "home environment" OR "community setting*" OR "community-dwelling" OR "habitual environment"))* |
|  | AND | *DOCTYPE(ar)* |
| **Web of Science** |  | *TS=(("Parkinson disease" OR "Parkinsonian disorder*" OR parkinson* OR parkinsonian OR parkinsonism OR "Lewy body parkinson*" OR PD OR stroke OR strokes OR poststroke OR "post-stroke" OR "post stroke" OR "cerebrovascular accident*" OR "brain infarction" OR "cerebral infarction")* |
|  | AND | *(gait OR walk* OR ambulation OR ambulat* OR locomot* OR stride* OR "walking speed" OR "gait speed" OR "step length" OR "step time" OR "stride length" OR "stride time" OR spatiotemporal OR "gait parameter*" OR "gait characteristic*" )* |
|  | AND | *("wearable electronic device*" OR "wearable device*" OR "wearable technolog*" OR "wearable sensor*" OR "wearable system*" OR "body-worn sensor*" OR "body-worn device*" OR "body worn sensor*" OR "body worn device*" OR "inertial sensor*" OR "inertial measurement unit*" OR IMU OR IMUs OR acceleromet* OR gyroscop* OR magnetomet* OR "motion sensor*")* |
|  | AND | *("daily life" OR "daily living" OR "everyday life" OR "usual environment" OR "real world" OR "real-world" OR "free living" OR "free-living" OR unsupervised OR "remote monitoring" OR "ambulatory monitoring" OR "home environment" OR "community setting*" OR "community-dwelling" OR "habitual environment"))* |
|  | AND | *DT=(Article)* |
