# Supplementary material for: Wearable Sensors in Gait Assessment for Parkinson Disease and Stroke in Real-World Environments: Systematic Review and Meta-Analysis
Source: J Med Internet Res. 2026 Sep 25;28:e93413. doi: 10.2196/93413 (PMC13613968; doi:10.2196/93413)
Supplement: Supplementary file 2 [file jmir-v28-e93413-s002.docx]

(a) Quality checklist

**Reporting**

1. Is the hypothesis/aim/objective of the study clearly described? (1 point)
2. Are the main outcomes to be measured clearly described in the Introduction or Methods section? (1 point). If the main outcomes are first mentioned in the Results, reply 'No'.
3. Is the protocol clearly described? (1 points) The description should include the eight following elements: duration of the observation (days/hours), type of observation (e.g., partially scripted), type of activity (e.g., diurnal, nocturnal, weekdays), number of sensors, sensor type, sensor frequency, sensor position, and attachment (e.g., belt). Answer ‘No’ if two or more elements are missing, ‘Partially’ if one element is missing, and ‘Yes’ if the eight elements are specified.
4. Are walking bout detection and step detection methods clearly described or referred to? (1 point). Walk detection refers to the detection of walking bouts (required for ambulatory studies or when parameters are computed on the whole signal, but the whole time of the walk is included in the processing). Step detection includes the detection of all steps. The description should allow for answering the following questions: (1) Which signal and axis is analyzed (accelerometer, gyroscope)? (2) which detection method is used? Answer ‘No’ if the elements are not well described, ‘Partially’ if one element is not well described, and ‘Yes’ if all elements are well described.
5. Are the aggregation methods included in the analysis clearly specified? (1 point). Aggregated methods (e.g., total number of steps, daily average walking speed) should be specified in the methods section.
6. Are the characteristics of the patients included in the study clearly described? (1 point) In cohort studies and trials, the inclusion and/or exclusion criteria should be given. In case-control studies, a case definition and the source for controls should be given.
7. Are all gait-derived parameters used in the study defined in the methods section? (1 points) Definitions or citations should be reported.
8. Are the main findings of the study clearly described? (1 point) When they exist, quantitative findings should be reported in the Results section and discussed in the Discussion section for the main findings.
9. Does the study provide estimates of the random variability in the data for the main outcomes? (1 point) For non-normally distributed data, the interquartile range should be reported. For normally distributed data, the standard error, standard deviation or confidence intervals should be reported. If the distribution of the data is not described, it must be assumed that the estimates used were appropriate, and the item should be scored 1.
10. Have actual probability values been reported (e.g., 0.035 rather than <0.05) for the main outcomes (when significant) except where the probability value is less than 0.001%? (1 point) When no result is significant, the item is not quoted.

**External validity**

11. Were the subjects asked to participate in the study representative of the entire population from which they were recruited? (1 point) The study must identify the source population for patients and describe how the patients were selected. Patients will be representative if they comprise the entire source population, an unselected sample of consecutive patients, or a random sample. Random sampling is only feasible when a list of all members of the relevant population exists. When a study does not report the proportion of the source populations from which the patients are derived, the score should be 0 (unable to determine).
12. Were those subjects who were prepared to participate representative of the entire population from which they were recruited? (1 points) The proportion of those asked who agreed should be stated. Validation that the sample was representative would include demonstrating that the distribution of the main confounding factors was the same in the study sample and the source population.
13. Was there validation of the sensor used? (1 point) If the sensor is self-made and no analysis of reliability was evident in the article or a previous article, the score should be 0.
14. Were gait detection algorithms validated in the studied population (1 point).

**Internal validity**

15. If any of the results of the study were based on “data dredging”, was this made clear? (1 point)
16. Any analyses that were not planned at the outset of the study should be clearly indicated. If no retrospective unplanned subgroup analyses were reported, then score 1.
17. Were the statistical tests used to assess the main outcomes appropriate? (1 point). If no test for normality or no post-hoc corrections were performed when needed, only 1/2 point should be given. If both criteria fail, the score should be 0.
18. Were the main outcome measures used accurate (valid and reliable)? (1 point). For studies for which the outcome measures are clearly described, the score should be 1. For studies that refer to other work or demonstrate that the outcome measures are accurate, the score should be 1.
(b) Calculation of quality score

Studies are assessed based on the sum of scores for all 18 items, divided by the maximum possible score (18) and multiplied by 100 to yield a percentage score that represents the overall methodological quality of the manuscript.

Calculation for quality score:

| Reporting | / 10 * 100 | % |
| --- | --- | --- |
| External validity | / 4 * 100 | % |
| Internal validity | / 4 * 100 | % |
| Total score | / 18 * 100 | % |

| **Overall Quality Rating** | |
| --- | --- |
| *Quality Score* | *Quality Assessment* |
| 0 - 25% | Very Low |
| 25.1 - 50% | Low |
| 50.1 - 75% | Moderate |
| 75.1 - 100% | High |

(c) Quality score per report

| Reports | Reporting | External validity | Internal validity | Total quality score |
| --- | --- | --- | --- | --- |
| Parkinson’s disease | | | | |
| Weiss et al. 2014 [70] | 90 | 50 | 100 | 83.3 |
| Weiss et al. 2015 [71] | 90 | 50 | 100 | 83.3 |
| Weiss et al. 2015 [73] | 75 | 50 | 87.5 | 72.2 |
| Toosizadeh et al. 2015 [72] | 85 | 25 | 100 | 75 |
| Del Din et al. 2016 [67] | 100 | 50 | 87.5 | 86.1 |
| Mancini et al. 2018 [69] | 95 | 25 | 75 | 75 |
| Del Din et al. 2019 [44] | 100 | 50 | 100 | 88.9 |
| Galperin et al. 2019 [68] | 90 | 25 | 100 | 77.8 |
| Del Din et al. 2020 [43] | 95 | 50 | 100 | 86.1 |
| Terashi et al. 2020 [54] | 95 | 25 | 87.5 | 77.8 |
| Coates et al. 2020 [53] | 100 | 50 | 100 | 88.9 |
| Shah et al. 2020 [55] | 100 | 50 | 100 | 88.9 |
| Shah et al. 2020 [61] | 100 | 50 | 100 | 88.9 |
| Shah et al. 2020 [62] | 100 | 50 | 100 | 88.9 |
| Bouça-Machado et al. 2021 [56] | 95 | 75 | 87.5 | 88.9 |
| Ullrich et al. 2021 [57] | 95 | 50 | 100 | 86.1 |
| Adams et al. 2021 [49] | 95 | 25 | 75 | 75 |
| Atrsaei et al. 2021 [48] | 90 | 50 | 87.5 | 80.6 |
| Atrsaei et al. 2021 [74] | 100 | 50 | 87.5 | 86.1 |
| Corrà et al. 2021 [63] | 85 | 50 | 87.5 | 77.8 |
| Rehman et al. 2022 [50] | 95 | 100 | 87.5 | 94.4 |
| Roth et al. 2022 [51] | 95 | 50 | 75 | 80.6 |
| Ullrich et al. 2023 [58] | 95 | 50 | 100 | 86.1 |
| Caballo et al. 2023 [59] | 80 | 37.5 | 75 | 69.4 |
| Cohen et al. 2023 [60] | 95 | 50 | 87.5 | 83.3 |
| Salis et al. 2023 [24] | 95 | 50 | 100 | 86.1 |
| Mico‑Amigo et al. 2023^7^ [52] | 95 | 50 | 100 | 86.1 |
| Kirk et al. 2023 [28] | 100 | 50 | 87.5 | 86.1 |
| Romijnders et al. 2023 [26] | 90 | 100 | 100 | 94.4 |
| Shah et al. 2023 [75] | 95 | 50 | 87.5 | 83.3 |
| Mirelman et al. 2023 [64] | 95 | 50 | 87.5 | 83.3 |
| Kirk et al. 2024 [27] | 100 | 50 | 100 | 88.9 |
| Zampogna et al. 2024 [65] | 100 | 37.5 | 75 | 80.6 |
| Nishi et al. 2024 [66] | 95 | 50 | 87.5 | 83.3 |
| Kirk et al. 2026 [76] | 100 | 50 | 75 | 83.3 |
| Sidoroff et al. 2026 [77] | 90 | 50 | 100 | 83.3 |
| Yarnall et al. 2026 [78] | 90 | 50 | 87.5 | 80.6 |
| **Stroke** | | | | |
| Sanchez et al. 2015 [79] | 85 | 25 | 100 | 75 |
| Punt et al. 2016 [80] | 100 | 50 | 87.5 | 86.1 |
| Moore et al. 2017 [25] | 95 | 50 | 87.5 | 83.3 |
| Punt et al. 2017 [81] | 95 | 50 | 87.5 | 83.3 |
| Van de Port et al. 2020 [82] | 100 | 75 | 87.5 | 91.7 |
| Felius et al. 2025 [83] | 85 | 25 | 87.5 | 72.2 |

References

24.	Salis F, Bertuletti S, Bonci T, Caruso M, Scott K, Alcock L, et al. A multi-sensor wearable system for the assessment of diseased gait in real-world conditions. Front. Bioeng. Biotechnol. 2023;11:1143248. doi: 10.3389/fbioe.2023.1143248

25.	Moore SA, Hickey A, Lord S, Del Din S, Godfrey A, Rochester L. Comprehensive measurement of stroke gait characteristics with a single accelerometer in the laboratory and community: a feasibility, validity and reliability study. J NeuroEngineering Rehabil 2017;14(1):130. doi: 10.1186/s12984-017-0341-z

26.	Romijnders R, Salis F, Hansen C, Küderle A, Paraschiv-Ionescu A, Cereatti A, et al. Ecological validity of a deep learning algorithm to detect gait events from real-life walking bouts in mobility-limiting diseases. Front. Neurol. 2023;14:1247532. doi: 10.3389/fneur.2023.1247532

27.	Kirk C, Küderle A, Micó-Amigo ME, Bonci T, Paraschiv-Ionescu A, Ullrich M, et al. Mobilise-D insights to estimate real-world walking speed in multiple conditions with a wearable device. Sci Rep 2024;14(1):1754. doi: 10.1038/s41598-024-51766-5

28.	Kirk C, Zia Ur Rehman R, Galna B, Alcock L, Ranciati S, Palmerini L, et al. Can Digital Mobility Assessment Enhance the Clinical Assessment of Disease Severity in Parkinson’s Disease? JPD 2023;13(6):999–1009. doi: 10.3233/JPD-230044

43.	Del Din S, Galna B, Lord S, Nieuwboer A, Bekkers EMJ, Pelosin E, et al. Falls Risk in Relation to Activity Exposure in High-Risk Older Adults. The Journals of Gerontology: Series A 2020;75(6):1198–1205. doi: 10.1093/gerona/glaa007

44.	Del Din S, Galna B, Godfrey A, Bekkers EMJ, Pelosin E, Nieuwhof F, et al. Analysis of Free-Living Gait in Older Adults With and Without Parkinson’s Disease and With and Without a History of Falls: Identifying Generic and Disease-Specific Characteristics. The Journals of Gerontology: Series A 2019;74(4):500–506. doi: 10.1093/gerona/glx254

48.	Atrsaei A, Corrà MF, Dadashi F, Vila-Chã N, Maia L, Mariani B, et al. Gait speed in clinical and daily living assessments in Parkinson’s disease patients: performance versus capacity. npj Parkinsons Dis. 2021;7(1):24. doi: 10.1038/s41531-021-00171-0

49.	Adams JL, Dinesh K, Snyder CW, Xiong M, Tarolli CG, Sharma S, et al. A real-world study of wearable sensors in Parkinson’s disease. npj Parkinsons Dis. 2021;7(1):106. doi: 10.1038/s41531-021-00248-w

50.	Rehman RZU, Guan Y, Shi JQ, Alcock L, Yarnall AJ, Rochester L, et al. Investigating the Impact of Environment and Data Aggregation by Walking Bout Duration on Parkinson’s Disease Classification Using Machine Learning. Front. Aging Neurosci. 2022;14:808518. doi: 10.3389/fnagi.2022.808518

51.	Roth N, Ullrich M, Kuderle A, Gladow T, Marxreiter F, Gassner H, et al. Real-World Stair Ambulation Characteristics Differ Between Prospective Fallers and Non-Fallers in Parkinson’s Disease. IEEE J. Biomed. Health Inform. 2022;26(9):4733–4742. doi: 10.1109/JBHI.2022.3186766

52.	Micó-Amigo ME, Bonci T, Paraschiv-Ionescu A, Ullrich M, Kirk C, Soltani A, et al. Assessing real-world gait with digital technology? Validation, insights and recommendations from the Mobilise-D consortium. J NeuroEngineering Rehabil 2023;20(1):78. doi: 10.1186/s12984-023-01198-5

53.	Coates L, Shi J, Rochester L, Del Din S, Pantall A. Entropy of Real-World Gait in Parkinson’s Disease Determined from Wearable Sensors as a Digital Marker of Altered Ambulatory Behavior. Sensors 2020;20(9):2631. doi: 10.3390/s20092631

54.	Terashi H, Taguchi T, Ueta Y, Okubo Y, Mitoma H, Aizawa H. Analysis of non-invasive gait recording under free-living conditions in patients with Parkinson’s disease: relationship with global cognitive function and motor abnormalities. BMC Neurol 2020;20(1):161. doi: 10.1186/s12883-020-01729-w

55.	Shah VV, McNames J, Mancini M, Carlson-Kuhta P, Spain RI, Nutt JG, et al. Laboratory versus daily life gait characteristics in patients with multiple sclerosis, Parkinson’s disease, and matched controls. J NeuroEngineering Rehabil 2020;17(1):159. doi: 10.1186/s12984-020-00781-4

56.	Bouça-Machado R, Branco D, Fonseca G, Fernandes R, Abreu D, Guerreiro T, et al. Kinematic and Clinical Outcomes to Evaluate the Efficacy of a Multidisciplinary Intervention on Functional Mobility in Parkinson’s Disease. Front. Neurol. 2021;12:637620. doi: 10.3389/fneur.2021.637620

57.	Ullrich M, Mucke A, Kuderle A, Roth N, Gladow T, Gabner H, et al. Detection of Unsupervised Standardized Gait Tests From Real-World Inertial Sensor Data in Parkinson’s Disease. IEEE Trans. Neural Syst. Rehabil. Eng. 2021;29:2103–2111. doi: 10.1109/TNSRE.2021.3119390

58.	Ullrich M, Roth N, Kuderle A, Richer R, Gladow T, Gasner H, et al. Fall Risk Prediction in Parkinson’s Disease Using Real-World Inertial Sensor Gait Data. IEEE J. Biomed. Health Inform. 2023;27(1):319–328. doi: 10.1109/JBHI.2022.3215921

59.	Caballol N, Bayés À, Prats A, Martín-Baranera M, Quispe P. Feasibility of a wearable inertial sensor to assess motor complications and treatment in Parkinson’s disease. PLoS ONE 2023;18(2):e0279910. doi: 10.1371/journal.pone.0279910

60.	Cohen M, Herman T, Ganz N, Badichi I, Gurevich T, Hausdorff JM. Multidisciplinary Intensive Rehabilitation Program for People with Parkinson’s Disease: Gaps between the Clinic and Real-World Mobility. IJERPH 2023;20(5):3806. doi: 10.3390/ijerph20053806

61.	Shah VV, McNames J, Harker G, Mancini M, Carlson-Kuhta P, Nutt JG, et al. Effect of Bout Length on Gait Measures in People with and without Parkinson’s Disease during Daily Life. Sensors 2020;20(20):5769. doi: 10.3390/s20205769

62.	Shah VV, McNames J, Mancini M, Carlson-Kuhta P, Nutt JG, El-Gohary M, et al. Digital Biomarkers of Mobility in Parkinson’s Disease During Daily Living. JPD 2020;10(3):1099–1111. doi: 10.3233/JPD-201914

63.	Corrà MF, Atrsaei A, Sardoreira A, Hansen C, Aminian K, Correia M, et al. Comparison of Laboratory and Daily-Life Gait Speed Assessment during ON and OFF States in Parkinson’s Disease. Sensors 2021;21(12):3974. doi: 10.3390/s21123974

64.	Mirelman A, Volkov J, Salomon A, Gazit E, Nieuwboer A, Rochester L, et al. Digital Mobility Measures: A Window into Real‐World Severity and Progression of Parkinson’s Disease. Movement Disorders 2024;39(2):328–338. doi: 10.1002/mds.29689

65.	Zampogna A, Borzì L, Rinaldi D, Artusi CA, Imbalzano G, Patera M, et al. Unveiling the Unpredictable in Parkinson’s Disease: Sensor-Based Monitoring of Dyskinesias and Freezing of Gait in Daily Life. Bioengineering 2024;11(5):440. doi: 10.3390/bioengineering11050440

66.	Nishi Y, Fujii S, Ikuno K, Terasawa Y, Morioka S. Adjustability of Gait Speed in Clinics and Free-Living Environments for People With Parkinson’s Disease. JMD 2024;17(4):416–424. doi: 10.14802/jmd.24167

67.	Del Din S, Godfrey A, Galna B, Lord S, Rochester L. Free-living gait characteristics in ageing and Parkinson’s disease: impact of environment and ambulatory bout length. J NeuroEngineering Rehabil 2016;13(1):46. doi: 10.1186/s12984-016-0154-5

68.	Galperin I, Hillel I, Del Din S, Bekkers EMJ, Nieuwboer A, Abbruzzese G, et al. Associations between daily-living physical activity and laboratory-based assessments of motor severity in patients with falls and Parkinson’s disease. Parkinsonism & Related Disorders 2019;62:85–90. doi: 10.1016/j.parkreldis.2019.01.022

69.	Mancini M, Curtze C, Stuart S, El-Gohary M, McNames J, Nutt JG, et al. The Impact Of Freezing Of Gait On Balance Perception And Mobility In Community-Living With Parkinson’S Disease. 2018 40th Annual International Conference of the IEEE Engineering in Medicine and Biology Society (EMBC). July 2018; Honolulu, HI, USA. Honolulu, HI, USA: IEEE; 2018. p. 3040–3043. doi: 10.1109/EMBC.2018.8512910

70.	Weiss A, Herman T, Giladi N, Hausdorff JM. Objective Assessment of Fall Risk in Parkinson’s Disease Using a Body-Fixed Sensor Worn for 3 Days. PLoS ONE 2014;9(5):e96675. doi: 10.1371/journal.pone.0096675

71.	Weiss A, Herman T, Giladi N, Hausdorff JM. Association between Community Ambulation Walking Patterns and Cognitive Function in Patients with Parkinson’s Disease: Further Insights into Motor-Cognitive Links. Parkinson’s Disease 2015;2015:1–11. doi: 10.1155/2015/547065

72.	Toosizadeh N, Mohler J, Lei H, Parvaneh S, Sherman S, Najafi B. Motor Performance Assessment in Parkinson’s Disease: Association between Objective In-Clinic, Objective In-Home, and Subjective/Semi-Objective Measures. PLoS ONE 2015;10(4):e0124763. doi: 10.1371/journal.pone.0124763

73.	Weiss A, Herman T, Giladi N, Hausdorff JM. New evidence for gait abnormalities among Parkinson’s disease patients who suffer from freezing of gait: insights using a body-fixed sensor worn for 3 days. J Neural Transm 2015;122(3):403–410. doi: 10.1007/s00702-014-1279-y

74.	Atrsaei A, Hansen C, Elshehabi M, Solbrig S, Berg D, Liepelt-Scarfone I, et al. Effect of Fear of Falling on Mobility Measured During Lab and Daily Activity Assessments in Parkinson’s Disease. Front. Aging Neurosci. 2021;13:722830. doi: 10.3389/fnagi.2021.722830

75.	Shah VV, McNames J, Carlson‐Kuhta P, Nutt JG, El‐Gohary M, Sowalsky K, et al. Effect of Levodopa and Environmental Setting on Gait and Turning Digital Markers Related to Falls in People with Parkinson’s Disease. Movement Disord Clin Pract 2023;10(2):223–230. doi: 10.1002/mdc3.13601

76.	Kirk C, Rehman RZU, Galna B, Ranciati S, Packer E, Ireson N, et al. Toward an understanding of real-world mobility in Parkinson’s: insights from enhanced contextualisation using GPS-derived location and data-driven modeling of walking speed. Front. Aging Neurosci. 2026;18:1746429. doi: 10.3389/fnagi.2026.1746429

77.	Sidoroff V, Moradi H, Prigent G, Jagusch F, Teckenburg I, Asalian M, et al. Moving beyond the hospital: in-depth characterization of daily-life mobility in patients with atypical Parkinsonian disorders. npj Parkinsons Dis. 2026;12(1):34. doi: 10.1038/s41531-025-01242-2

78.	Yarnall AJ, Alcock L, Schlenstedt C, Armengol C, Becker C, Brown P, et al. The future of clinical trials? Monitoring mobility as an outcome measure in Parkinson’s disease: The mobilise-D study. Journal of Parkinson’s Disease 2026;16(1):110–124. doi: 10.1177/1877718X251407026

79.	Sanchez M, Bussmann J, Janssen W, Horemans H, Chastin S, Heijenbrok M, et al. Accelerometric assessment of different dimensions of natural walking during the first year after stroke: Recovery of amount, distribution, quality and speed of walking. J Rehabil Med 2015;47(8):714–721. doi: 10.2340/16501977-1994

80.	Punt M, Bruijn SM, Van Schooten KS, Pijnappels M, Van De Port IG, Wittink H, et al. Characteristics of daily life gait in fall and non fall-prone stroke survivors and controls. J NeuroEngineering Rehabil 2016;13(1):67. doi: 10.1186/s12984-016-0176-z

81.	Punt M, Bruijn S, Wittink H, Port I, Dieen J. Do clinical assessments, steady-state or daily-life gait characteristics predict falls in ambulatory chronic stroke survivors? J Rehabil Med 2017;49(5):402–409. doi: 10.2340/16501977-2234

82.	Van De Port I, Punt M, Meijer JW. Walking activity and its determinants in free-living ambulatory people in a chronic phase after stroke: a cross-sectional study. Disability and Rehabilitation 2020;42(5):636–641. doi: 10.1080/09638288.2018.1504327

83.	Felius RAW, Punt M, Wouda NC, Geerars M, Bruijn SM, Van Dieën JH. Predicting community walking after stroke. Front. Stroke 2025;4:1523242. doi: 10.3389/fstro.2025.1523242
